# Impact of child emotional and behavioural difficulties on educational outcomes of primary school children in Ethiopia: a population-based cohort study

**DOI:** 10.1186/s13034-020-00326-6

**Published:** 2020-05-16

**Authors:** Habtamu Mekonnen, Girmay Medhin, Mark Tomlinson, Atalay Alem, Martin Prince, Charlotte Hanlon

**Affiliations:** 1 Department of Psychiatry, School of Medicine, College of Health Sciences, Addis Ababa University, Addis Ababa, Ethiopia; 2 Department of Psychology, College of Education and Behavioural Sciences, Jimma University, Jimma, Ethiopia; 3 Aklilu-Lemma Institute of Pathobiology, Addis Ababa University, Addis Ababa, Ethiopia; 4 Department of Psychology, Stellenbosch University, Stellenbosch, South Africa; 5 Health Service and Population Research Department, Centre for Global Mental Health, Institute of Psychiatry, Psychology and Neuroscience, King’s College London, London, UK; 6 Centre for Innovative Drug Development and Therapeutic Trials for Africa (CDT-Africa), College of Health Sciences, Addis Ababa University, Addis Ababa, Ethiopia

**Keywords:** Child education, Absenteeism, Child emotional and behavioral difficulty, Child mental health, Cohort study, Sub-saharan Africa

## Abstract

**Background:**

The relationship between child emotional and behavioural difficulties (EBD) and educational outcomes has not been investigated in prospective, community studies from low-income countries.

**Methods:**

The association between child EBD symptoms and educational outcomes was examined in an ongoing cohort of 2090 mother–child dyads. Child EBD was measured when the mean age of children was 6.5 years, SD 0.04 (T0) and 8.4, SD 0.5 years (T1) using the Strength and Difficulties Questionnaire (SDQ). Educational outcomes were obtained from maternal report (drop-out) at T1 and from school records at when the mean age of the children was 9.3 (SD 0.5) years (T2).

**Result:**

After adjusting for potential confounders, child EBD symptoms at T1 were associated significantly with school absenteeism at T2: SDQ total score: Risk Ratio (RR) 1.01; 95% confidence interval (CI) 1.01, 1.02; SDQ high score (≥ 14) RR 1.36; 95% CI 1.24, 1.48; emotional subscale RR 1.03; 95% CI 1.01, 1.04; hyperactivity subscale RR 1.03; 95% CI 1.02, 1.04 and peer problems subscale (RR 1.02; 95% CI 1.00, 1.04). High SDQ (β = − 2.89; 95% CI − 5.73, − 0.06) and the conduct problems sub-scale (β = − 0.57; 95% CI − 1.02, − 0.12) had a significant negative association with academic achievement. There was no significant association between child EBD and school drop-out.

**Conclusion:**

Prospective associations were found between child EBD symptoms and increased school absenteeism and lower academic achievement, suggesting the need for child mental health to be considered in interventions targeting improvement of school attendance and academic achievement in low-income countries.

## Background

Early experiences play an important role in the later life of a child, including their educational success [1, 2]. One crucial, but often overlooked, aspect of holistic child development is the mental health of the child. In studies from high income countries, child emotional and behavioural disorder (EBD) symptoms are associated with poorer social adaptation [3], compromised physical wellbeing, functional impairment and worse educational outcomes [4, 5]. The fourth United Nations Sustainable Development Goal (SDG) aims for inclusive and quality education for all children [6], an aspiration unlikely to be achieved without due attention being given to child mental health.

Most studies from LMICs have focused on physical rather than mental health in relation to child educational outcomes [7, 8]. We identified just one prospective study examining the impact of child EBD on educational outcomes, from Chile, an upper middle income country [9]. In studies from Nigeria [10], Pakistan [11] and Sri Lanka [12], child EBD symptoms were associated with retrospectively-ascertained educational outcomes, but the direction of the association could not be examined. To the best of our knowledge, there have been no prospective studies examining the association between child EBD and education outcomes from a low income country, where the impact of poverty on school attendance and academic achievement may overwhelm any contribution of child EBD symptoms.

In this study, we examined prospectively the association between child EBD symptoms and educational outcomes across two waves of a population-based cohort study in rural Ethiopia. We hypothesised that children with symptoms of EBD at pre- and early school age would be at increased risk of school absenteeism, drop-out and poorer academic achievement over 2 years of follow-up.

## Methodology

### Study design

The study was an extension of a population-based birth cohort, the child outcomes in relation to maternal mental illness in Ethiopia (C-MaMiE) study [13]. For the current analysis, three contiguous birth cohorts were included: the original C-MaMiE cohort, cohort A (born in the preceding 12 months) and cohort B (born in the following 12 months). At assessment time-point 0 (T0), children were all aged 6.5 years (SD 0.04) and then followed up at two further assessment time-points (T1 and T2) for an average total period of 2.2 to 3.5 years, depending upon the birth cohort. The prospective relationship between the primary exposure and outcomes was assessed separately for two waves of the cohort: from T0 to T1 (maternal report of drop-out) and from T1 to T2 (school records of drop-out, absenteeism and achievement).

### Study setting

The C-MaMiE study is located within the Health and Demographic Surveillance Site (HDSS) of the Butajira Rural Health programme, established in 1986 [14]. The Butajira HDSS includes nine rural administrative sub-districts of different ecological zones and one urban sub-district in Butajira Town. Butajira is a predominantly rural area found in the Gurage Zone of the Southern Nations, Nationalities and Peoples’ Region (SNNPR) of Ethiopia, 135 km away from the capital city, Addis Ababa. The Zone is characterised by high population density with substantial ethnic and linguistic diversity. The local economy is based on mixed farming of cash crops (khat and chilli peppers) and staples (maize and “false banana” or *Ensete ventricosun).*

### Educational context

In Ethiopia there is no school fee for governmental schools; however, parents are expected to cover costs for school uniform, exercise books and food [15]. In the study area, less than 0.3% of the study children attend private schools. The country has no standardized measure of school readiness but depends on self-reported age of 7 years. Primary education has two cycles; basic (grades 1–4) and general (grades 5–8) between the ages 7 and 14 years [15]. Coverage of primary school was estimated to be 85.5%, with 10.1% drop-out overall, but higher drop-out in grade one (16.8%) and 6.7% grade repetition in 2015/2016 [16]. Except for two national examinations at the completion of grades 10 and 12, and one regional examination at completion of grade 8, the academic performance of students is assessed by the class teacher using non-standardized tests.

### Study participants

The original C-MaMiE cohort was established between July 2005 and February 2006 in the Butajira HDSS [13]. At recruitment, inclusion criteria for the women were: age between 15 and 49 years, ability to speak Amharic, resident of the HDSS and in the third trimester of pregnancy. Fewer than 3% of eligible women were excluded at recruitment because of lack of fluency in Amharic language. A total of 1065 out of 1234 eligible women (86.3%) were recruited and followed to date. When the C-MaMiE children were aged 6.5 years, the cohort size was augmented using the HDSS to identify cohort A (n = 543; 94.9% of eligible) and cohort B (n = 717; 92.8% of eligible) with application of identical inclusion criteria.

### Assessment time-points

*Exposures* assessed at T0 (2012/2013 academic year) and T1 (2013/2014 academic year).

*Outcomes* assessed at T1 and T2 (2014/2015 academic year) at a mean age of 9.3 (SD 0.5) years. See Additional file 1 for a graphical depiction of the cohort waves and mean age for each cohort.

### Measures

#### Outcomes

*Absenteeism* The total number of days of absence was obtained from the daily school attendance record at T2.

*School drop-out* This was operationalised as the proportion of students who had enrolled at the beginning of the academic year (September) but who had dropped out of school before the end of the academic year (June) and obtained from maternal report at T1 and from school records at T2. Presumed reasons for drop-out at T1 were also obtained from mothers. Children who drop out of school at T1 can be re-enroled and, therefore, drop-out again at T2.

*Academic achievement* The averaged grade point over two semesters of the Ethiopian school year was measured at T2. The class teacher grading of academic achievement is non-standardised and incorporates mastery of content, class participation and interaction, conduct, homework, progress over time and school attendance.

#### Primary exposure

*Child EBD* was measured at T0 and T1 using the brief screening, parent-report version of the Strengths and Difficulties Questionnaire (SDQ) for emotional and behavioural difficulties in children and adolescents aged 4 to 16 years [17]. An approved Amharic version of the SDQ is available [18]. Within the C-MaMiE cohort, the SDQ has been found to have construct and convergent validity when used as a continuous scale [19]. In keeping with the recommendation of the developer, we applied a score of 14 and above to indicate high emotional and behavioural symptoms, but we were not able to relate this to mental health problems as the criterion validity of the SDQ against clinical diagnoses has not been established in Ethiopia.

#### Potential confounding factors

All potential confounders were assessed at the time-point preceding the outcome measure (T0 for T1 and T1 for T2).

*Maternal common mental disorder* was measured using the World Health Organization (WHO) 20-item version of the Self-Reporting Questionnaire (SRQ-20) [20] which has been validated in this setting [13].

*Stressful life events* over the preceding 6 months were measured using an adapted version of the List of Threatening Experiences [21].

*Socio-economic status (SES)* was measured using self-report of house roof composition (corrugated iron vs. thatched), the experience of hunger in the preceding month, and the existence of emergency resources for times of crisis.

*Paternal substance use* report of the frequency of alcohol or khat use by the father was obtained from maternal self-report.

*Child nutritional status* height was assessed by project data collectors using a portable stadiometer with a movable head piece for height. Using the World Health Organization (WHO) reference population [22], height-for-age z scores were calculated with the WHO Anthro software [23]. Although weight was also assessed, height-for-age and weight-for-age were collinear. As height-for-age is a cumulative indicator of nutritional status [24], the height-for-age z scores were preferred for the analysis.

*Demographic characteristics* age of the mother, marital status, literacy level, birth order and sex of the child were obtained from self-report of the mother.

#### Data management

*Data collection procedures* interviews with the women and anthropometric measures of the child were carried out in the woman’s home, or in the surrounding area, according to their preference. Child anthropometric measures were also conducted in school when convenient. The project data collectors, who had all completed secondary school education and above, were given 3 days of intensive refresher training on the administration of instruments. The questionnaires were piloted before commencing data collection and discrepancies in ratings were discussed to ensure that the data collectors had a common understanding.

*Maintaining data quality* supervisors monitored data quality and identified missing data in the field. Random quality checks were performed on a sample of assessments. Data were double entered with EpiData [25] by project data clerks on the day of data collection, where possible.

### Statistical analyses

Stata software version 12 [26] was used for the analysis. The analysis was hypothesis-driven with potential confounders specified a priori. SDQ total score, SDQ high (≥ 14) score and total scores of SDQ sub-scales were the primary exposures. Logistic regression was used for school drop-out (binary outcomes), zero-inflated poisson regression was used to model absenteeism (count variable, with excess zeroes), and linear regression was used to model academic achievement (continuous outcome, normally distributed). Estimates of association were presented with their respective 95% confidence intervals. An exploratory analysis was carried out to examine whether there was effect modification by cohort between the primary exposure and outcomes.

## Results

**Fig. 1:**
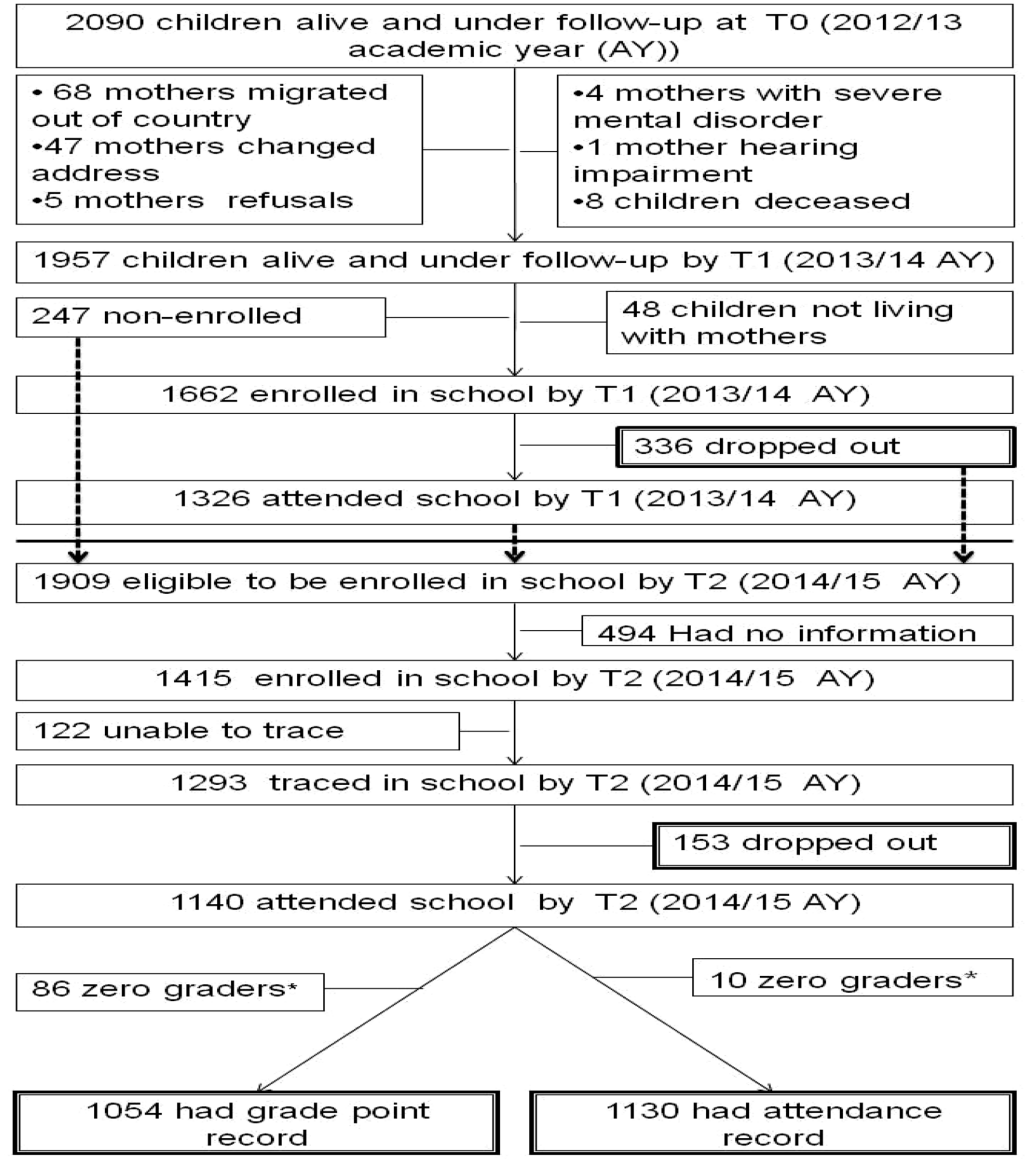
Follow-up chart. *Had no attendance and academic records, ^T0^assessment time-point 0, ^T1^assessment time-point 1, ^T2^assessment time-point 2

At T0 and T1, 2090 and 1957 mother–child dyads, respectively, were available for follow-up. See Fig. 1.

There was no difference between those remaining in the cohort and who were lost to follow-up from T0 to T1 and from T0 to T2 in terms of maternal CMD, parental literacy, socioeconomic status, EBD symptoms and birth order. However, those lost to follow-up were more likely to be boys and less likely to have emergency resources from T0 to T1 and those lost to follow-up were more likely to be stunted, more likely to have thatched roof cover and less likely to have emergency resources from T0 to T2. See Additional files 2 and 3.

The percentage of children who scored 14 and above on the SDQ was 7.2% (n = 151) at T0 and 5.9% (n = 112) at T1. At T0, 58.3% of high scorers were boys, which increased to 73.2% at T1. Boys scored higher than girls on all sub-scales of the SDQ except on the emotional sub-scale. In both boys and girls, symptoms of hyperactivity problems were most frequently noted, followed by peer relationship problems and conduct problems (see Additional file 4 for the graphic depiction).

School drop-out was 20.2% (n = 336) at T1 and 11.8% (n = 153) at T2. At T2, the median number of days of absence was 5 (interquartile range 2–11), with 191 (17.0%) children having no days of absence. The mean average grade point was 62.5 out of 100 (SD 9.2). See Tables 1 and 2.

**Table 1:** Summary of participants in relation to educational outcome at T1

Characteristics at T0	Child school drop-out at T1 (n = 1662)		MV
	Dropped out N (%)	Attending N (%)	
	336 (20.2)	1326 (79.8)	
Child EBD			
High SDQ (≥ 14)	21 (6.3)	95 (7.2)	14
Low SDQ (< 14)	311 (93.7)	1222 (92.8)	
Mean maternal age in years (standard deviation)	34.0 (6.0)	33.0 (6.0)	5
Maternal literacy			
Non-literate	307 (91.6)	1100 (83.3)	6
Literate	28 (8.4)	221 (16.7)	
Paternal literacy			
Non-literate	148 (46.7)	423 (33.3)	75
Literate	169 (53.3)	847 (66.7)	
Marital status			
Monogamous	263 (78.5)	1089 (82.4)	6
Polygamous	54 (16.1)	182 (13.8)	
Divorced, widowed, separated	18 (5.4)	50 (3.8)	
Had hunger in preceding month			
Yes	42 (12.5)	80 (6.1)	6
No	293 (87.5)	1241 (93.9)	
Had emergency resources			
No	164 (48.9)	485 (36.7)	6
Yes	171 (51.1)	836 (63.3)	
Roof material			
Thatched roof	242 (72.2)	809 (61.2)	6
Corrugated iron	93 (27.8)	512 (38.8)	
Father’s khat use			
Weekly	264 (83.5)	976 (76.9)	77
Less than weekly	52 (16.5)	293 (23.1)	
Father’s alcohol use			
Weekly	55 (17.4)	185 (14.6)	77
Less than weekly	261 (82.6)	1084 (85.4)	
Negative life event in the last 6 months			
No life event	272 (81.9)	1065 (81.2)	18
1 life event	48 (14.5)	184 (14.0)	
2 or more	12 (3.6)	63 (4.8)	
Maternal mental health			
SRQ score ≥ 6	19 (5.7)	54 (4.1)	6
SRQ score < 6	316 (94.3)	1267 (95.9)	
Childbirth order			
First	26 (7.7)	212 (16.0)	0
Middle or last	310 (92.3)	1114 (84.0)	
Sex of the child			
Boy	201 (59.8)	670 5(0.6)	0
Girl	135 (40.2)	654 (49.4)	
Child age mean standard deviations	6.5 (0.04)	6.5 (0.04)	0
Child nutritional status (height for age)			
Stunted	111 (33.4)	297 (46.0)	15
Non-stunted	221 (66.6)	1018 (77.4)	

*SDQ* Strength and Difficulty Questionnaire, *SRQ* Self Reporting Questionnaire, * MV* missing value, *T0* assessment time-point 0, *T1* assessment time-point 1

**Table 2:** Summary of participants in relation to educational outcomes at T2

Characteristics at T1	Outcomes measured at T2						
	Child school drop-out (n = 1293)		MV	absenteeism (n = 1130)	MV	Academic achievement (n = 1054)	MV
	Dropped out	Attending					
	N (%)	N (%)		Median (25th, 75th centile)		Mean (SD)	
	153 (11.8)	1140 (88.2)		5 (2, 11)		62.5 (9.2)	
Child EBD							
SDQ score (≥ 14)	11 (7.4)	58 (5.2)	18	5 (3, 12)	13	60.2 (6.3)	9
SDQ score < 14	138 (92.6)	1068 (94.8)		4 (2, 11)		62.6 (9.3)	
Mean maternal age in years (standard deviation)	33.9 (5.51)	33.5 (5.45)	7		2		6
Maternal literacy							
Non-literate	142 (93.4)	997 (88.0)	8	5 (2, 12)	6	62.5 (9.3)	6
Literate	10 (6.6)	136 (12.0)		3 (3, 5)		62.3 (8.6)	
Paternal literacy							
Non-literate	62 (42.5)	400 (36.8)	60	6 (3, 14)	52	61.3 (9.2)	50
Literate	84 (57.5)	687 (63.2)		4 (1, 9)		63.2 (9.2)	
Marital status							
Monogamous	116 (76.8)	908 (81.0)	21	5 (2, 12)	18	62.8 (9.3)	17
Polygamous	28 (18.5)	160 (14.3)		4 (2, 9)		61.1 (8.9)	
Divorced, widowed, separated	7 (4.6)	53 (4.7)		4 (1, 6)		62.1 (8.1)	
Had hunger in preceding month							
Yes	12 (7.9)	50 (4.4)	9	5 (2, 11)	7	62.1 (7.8)	7
No	140 (92.1)	1082 (95.6)		5 (2, 11)		62.5 (9.3)	
Had emergency resource							
No	49 (32.2)	322 (28.4)	8	5 (2, 11)	6	62.1 (9.1)	6
Yes	103 (67.8)	811 (71.6)		5 (2, 11)		62.7 (9.3)	
Roof material							
Thatched	94 (61.8)	601 (53.0)	8	6 (3, 14)	6	62.8 (9.7)	6
Corrugated iron	58 (38.2)	532 (47.0)		3 (1, 8)		62.2 (8.7)	
Father’s khat use							
Weekly	104 (72.2)	880 (82.7)	83	5 (2, 12)	75	62.3 (9.2)	58
Less than weekly	40 (27.8)	184 (17.3)		3.5 (0, 8)		63.6 (9.5)	
Father’s alcohol use							
Weekly	30 (20.8)	173 (16.2)	85	3.5 (1, 7)	73	64.6 (9.4)	60
Less than weekly	114 (79.2)	893 (83.8)		5 (2, 12)		62.2 (9.2)	
Negative life event in the last 6 months							
No life event	108 (71.1)	860 (75.9)	8	5 (2, 12)	6	62.5 (9.1)	6
1 life event	31 (20.4)	196 (17.3)		4 (2, 9)		62.8 (9.8)	
2 or more	13 (8.6)	77 (6.8)		3 (2, 8)		61.2 (8.2)	
Maternal CMD							
SRQ score ≥ 6	20 (13.2)	117 (10.3)	8	5 (2, 11)	6	62.0 (9.5)	6
SRQ score < 6	132 (86.8)	1016 (89.7)		5 (2, 11)		62.6 (9.2)	
Childbirth order							
First	16 (10.5)	156 (13.8)	0	4.5 (2, 14)	0	62.8 (8.9)	0
Middle or last	136 (89.5)	977 (86.2)		5 (2, 11)		62.5 (9.3)	
Sex of the child							
Boy	85 (55.6)	590 (52.0)	0	5 (2, 12)	0	62.6 (9.2)	0
Girl	68 (44.4)	544 (48.0)		4 (2, 10)		62.4 (9.2)	
Child nutritional status							
Stunted	53 (34.9)	244 (21.6)	11	5 (2, 10)	9	62.3 (9.5)	9
Non-stunted	99 (65.10	886 (78.4)		4.5 (2, 11)		62.6 (9.2)	

*SDQ* Strength and Difficulty Questionnaire, *SRQ * Self Reporting Questionnaire, *MV* missing value, *T1* assessment time-point 1, *T2* assessment time-point 2

The reasons given by mothers for their children dropping out of school at T1 are presented in Additional file 5, and included being bullied, disciplinary action and child school refusal. In the fully adjusted models, child EBD symptoms were not significantly associated with school drop-out at either T1 or T2. The total score on the SDQ (Risk Ratio = 1.01, CI (1.01, 1.02), p = < 0.001), emotional (Risk Ratio = 1.03, CI (1.01, 1.04, p = 0.001), hyperactivity (Risk Ratio = 1.03, CI (1.02, 1.04, p < 0.001), and peer relationship (Risk Ratio = 1.02, CI (1.00, 1.04, p = 0.022), sub-scales measured at T1 were significantly associated with absenteeism at T2. High SDQ (β coefficient = − 2.90 (− 5.76, − 0.05) p = 0.046), and conduct problem (β coefficient = − 0.55, CI (− 1.01, − 0.10) p = 0.017), sub-scale scores at T1 was significantly inversely associated with academic achievement at T2 (Table 3).

**Table 3:** Impact of child emotional and behavioural difficulties on educational outcomes

Exposures	Models	Child EBD at T0	Child EBD at T1		
		Drop-out at T1 (n = 1662) Odds ratio (95% confidence interval; CI)	Drop-out at T2 (n = 1293) Odds ratio (95% CI)	Absenteeism at T2 (n = 1130) Risk ratio (95% CI)	Academic achievement at T2 (n = 1054) β coefficient (95% CI)
SDQ sub-scales (total scores)					
Emotional problem	Crude	1.05 (0.96, 1.15)	1.06 (0.95, 1.19)	1.02 (1.00, 1.03)**	− 0.11 (− 0.51, 0.30)
	Adjusted^a^	1.03 (0.93, 1.13)	1.01 (0.89, 1.14)	1.03 (1.01, 1.04)**	− 0.13 (− 0.55, 0.31)
Conduct difficulty	Crude	1.10 (1.00, 1.20)**	1.05 (0.93, 1.18)	0.99 (0.97, 1.00)	− 0.41 (− 0.82, 0.00)**
	Adjusted^a^	1.05 (0.95, 1.16)	1.01 (0.88, 1.16)	1.01 (0.99, 1.02)	− 0.55 (− 1.01, − 0.10)**
Hyperactivity problem	Crude	1.05 (0.98, 1.12)	0.99 (0.91, 1.09)	1.02 (1.00, 1.03)**	− 0.15 (− 0.46, 0.16)
	Adjusted^a^	1.02 (0.94, 1.10)	0.98 (0.88, 1.109)	1.03 (1.02, 1.04)**	− 0.11 (− 0.45, 0.23)
Peer relationship problem	Crude	1.03 (0.94, 1.12)	0.99 (0.86, 1.14)	1.00 (0.98, 1.02)	− 0.21 (− 0.67, 0.25)
	Adjusted^a^	1.02 (0.93, 1.12)	0.97 (0.83, 1.13)	1.02 (1.00, 1.04)**	− 0.12 (− 0.63, 0.39)
SDQ full scale					
Total score	Crude	1.01 (0.98, 1.04)	1.01 (0.97, 1.05)	1.00 (1.00, 1.01)	− 0.09 (− 0.22, 0.04)
	Adjusted^a^	1.01 (0.97, 1.03)	0.99 (0.95, 1.04)	1.01 (1.01, 1.02)**	− 0.10 (− 0.24, 0.04)
High score (≥ 14)	Crude	0.87 (0.53, 1.41)	1.48 (0.75, 2.86)	1.23 (1.13, 1.34)**	− 2.44 (− 5.04, − 0.16)
	Adjusted^a^	0.71 (0.41, 1.23)	1.17 (0.56, 2.44)	1.36 (1.24, 1.48)**	− 2.90 (− 5.76, − 0.05)**

*T0* assessment time-point 0, *T1* assessment time-point 1, *T2* assessment time-point 2

** The association is significant at 95% CI and P value less than 0.05

^a^Maternal age, marital status, maternal and paternal level of literacy, SES, paternal substance use, negative life event, maternal CMD, child sex, birth order and nutritional status

There was some evidence of modification of the associations between EBD and absenteeism by cohort (tests for interaction: C-MaMiE cohort p = 0.034, cohort B p = 0.001). In the C-MaMiE cohort (n = 464) the associations between child EBD and absenteeism remained significant. In cohort A (n = 293), with the older children, the association became non-significant, and in cohort B (n = 367), with the younger children, the direction of association was reversed. See Additional file 6 for the full stratified analysis.

## Discussion

In this prospective cohort study from a rural population in Ethiopia, child EBD symptoms were associated significantly with child school absenteeism. High SDQ score and the SDQ conduct difficulty sub-scale were significantly associated with poor academic achievement. There was no association between child EBD symptoms and school drop-out.

To the best of our knowledge, this is the first study from sub-Saharan Africa to investigate the association between pre- and early school age child EBD symptoms and subsequent educational outcomes. Our study benefited from a large sample size, prospective study design, high follow-up rate and the use of culturally validated measures. There were also limitations. The measures of child EBD and maternal mental health were screening tools and do not represent a clinical level of difficulty. The measurement of child EBD symptoms relied upon the report of the mother. It would have been preferable to combine this with reports from the teachers. However, class sizes are large (average 63 per class in SNNPR) [16]. Therefore, teachers may not know individual children well enough to identify symptoms of EBD, except those behaviours leading to disciplinary challenges. We assessed academic achievement using non-standardized, composite assessments by individual teachers. Although this approach could have increased measurement error, it may be more valid than narrowly focused assessments of content mastery. As attendance is a component of the rating of academic achievement, there could have been overlap in these outcome measures. Routinely recorded attendance data to identify absenteeism may underestimate the true number of missed days. The indicators of socioeconomic status may not have been adequate, leading to the possibility of residual confounding. In addition, we may not have adjusted adequately for child physical ill-health, although we included anthropometric measures which are a proxy indicator of cumulative exposure to illness.

Our finding of an association between child EBD symptoms and subsequent absenteeism is in keeping with the large prospective study from Chile [9] and a retrospective study from Sri Lanka, where child mental health problems at the time of assessment were associated with absenteeism (dichotomised as > 20% of school days missed) in the preceding year [12]. In our study, the association between child EBD and absenteeism remained significant after adjusting for several indicators of social adversity, which are known to be strong predictors of school absenteeism and drop-out in rural low-income country settings [27]. There was some evidence of effect modification of the association between child EBD and absenteeism in our analyses, although the sub-samples were small. It is possible that the importance of child EBD symptoms in relation to absenteeism varies with the developmental stage of the child and the prevalence of other risk factors for absenteeism. We were not, however, able to investigate the possible mechanisms through which EBD symptoms are associated with absenteeism. In high-income countries, child EBD symptoms may negatively affect peer interactions, attitudes towards school, self-worth, and the responses of teachers and peers to the child [4]. In turn, this may negatively affect regular attendance. In low-income countries such as Ethiopia, where there is limited recognition of child EBD symptoms and scarce child mental health services these potential pathways from EBD to absenteeism may be amplified.

Absenteeism in a rural low-income country setting is likely to lead to poorer academic achievement due to the limited opportunities to catch up through independent study, given the lack of educational resources at home. Absenteeism is also on the pathway towards full drop-out from school [28]. However, in contrast to findings from Chile [9] and studies from high-income countries, e.g. [29], we did not find a significant association between child EBD symptoms and school drop-out. An association between child EBD and drop-out may have not been detected in our study because of the time for progression from absenteeism to drop-out and limited statistical power to detect an association. Nonetheless, the reasons given by women for their child dropping out of school at T1 included being bullied, disciplinary action and child school refusal, all of which suggest that child EBD symptoms may have had a role to play in school drop-out for some children.

The significant negative association of both high SDQ score and the SDQ conduct problems sub-scale with academic achievement in our study is also in line with the Chilean study [9], as well as the retrospective study from Nigeria [10] and a cross sectional study from Pakistan [11] and a review of studies from high-income countries [5]. We found different patterns of association between SDQ sub-scales and educational outcomes; while the conduct sub-scale was associated with academic achievement, emotional, peer relationship and hyperactivity sub-scales were associated with absenteeism. Conduct problems may be more easily recognised by teachers than internalising problems due to large class sizes and the nature of the problems. This may negatively affect the teacher’s subjective evaluation of academic performance which is based on a composite of participation, attendance and interaction. Emotional and peer relationship symptoms may play more of a role in absenteeism than academic achievement, which is also indicated by the parental report of bullying and school refusal as reasons for school drop-out. However, the interconnection between absenteeism, academic achievement and drop-out requires more detailed future study.

Child absenteeism is a barrier to the achievement of ‘inclusive and quality education for all children’, as articulated in the United Nations Sustainable Development Goal number 4. Recent recommendations from the Disease Control Priorities group for ‘best practice’ interventions at the community level for mental and neurological disorders in LMICs [30] included: socio-emotional learning, mental health awareness, detection of mental and neurological disorders in schools, early child enrichment or preschool educational and parenting programme for children. In Chile, providing students with school-based mental health services and skills for life [9] was associated with improvement in attendance and achievement. Earlier studies from Ethiopia have found food insecurity [27] and maternal mental health [31] to be important determinants of school attendance. Multi-faceted interventions may be required, which provide both social and psychological support to families of children who are absent from school, coupled with efforts to increase teacher and community awareness about mental health. Current efforts to scale-up mental health care in Ethiopia by integrating mental health into community health extension worker prevention and promotion activities, as well as into primary care services, [32] provide an important opportunity to improve child mental health and potentially impact on educational outcomes.

## Conclusions

In this study from rural Ethiopia, child emotional and behavioural difficulty symptoms were associated prospectively with absenteeism and negatively with academic achievement. We plan to follow-up the cohort to look at the changing patterns of EBD symptoms and longer-term impacts on standardised national examination performance. Interventions targeting reduction of school absenteeism and improvement of academic achievement need to attend to the mental health of the child.

## Supplementary information

**Additional file 1.** Assessments time-points and outcome measures.

**Additional file 2.** Difference beteen those children who remain and lost to follow-up from T0 to T1 (n = 2090).

**Additional file 3.** Difference beteen those children who do and do not have educational information at T1 (n = 1957).

**Additional file 4.** Graph of SDQ item frequency by sex of the child at T0.

**Additional file 5.** Maternal report of reasons for school drop-out at T1.

**Additional file 6.** Impact of child emotional and behavioural difficulties on absenteeism by cohort.

## Data Availability

The data are being used for a PhD student (HM) for his thesis and are not, therefore, available at the present time to the general public. The data may be requested from the corresponding author for verification of the analyses in this paper.

## Abbreviations

C-MaMiE: Child outcomes in relation to maternal mental illness in Ethiopia
EBD: Emotional and behavioural difficulty
HDSS: Health and Demographic Surveillance Site
LMICs: Low and middle income countries
RR: Risk ratio
SDG: Sustainable development goal (SDG)
SDQ: Strength and Difficulties Questionnaire
SNNRP: Southern Nations, Nationalities and Peoples’ Region
SRQ-20: Self-Reporting Questionnaire
WHO: World Health Organization
