# Supplementary material for: Impact of child emotional and behavioural difficulties on educational outcomes of primary school children in Ethiopia: a population-based cohort study
Source: Child Adolesc Psychiatry Ment Health. 2020 May 16;14:22. doi: 10.1186/s13034-020-00326-6 (PMC7231403; doi:10.1186/s13034-020-00326-6)
Supplement: Supplementary file 1 — Additional file 1. Assessments time-points and outcome measures. [file 13034_2020_326_MOESM1_ESM.doc]

# Additional file 1: Assessments time-points and outcome measures

|  | **Assessment time-points** | | | | | | | | | | | | |
| --- | --- | --- | --- | --- | --- | --- | --- | --- | --- | --- | --- | --- | --- |
|  | **T0^†^** | | | | | | | **T1^¥^** | | | **T2^₮^** | | |
|  | **Year** | **2011** | **2012** | | **2013** | **Mean age (SD^§^)**  **in year** | **Outcome measures** | **2014** | **Mean age (SD) in year** | **Outcome measures** | **2015** | **Mean age (SD**  **in year** | **Outcome measures** |
|  | **Months** | Aug-Dec | Jan- Oct | Nov-Dec | Jan-Aug |  |  | Apr-Oct |  |  | Jun -Aug |  |  |
| **Cohorts (with their ranges of birth date)** | **Cohort A**  (January 2005 to July 2005) |  |  |  |  | (6.5 (0.04) | None |  | 9.1 (0.2) | Drop-out |  | 10.0 (0.2) | Drop-out, absenteeism &academic achievement |
|  | **C-MaMiE Cohort**  (July 2005 to February 2006) |  |  |  |  | (6.5 (0.04) | None |  | 8.5 (0.3) | Drop-out |  | 9.3 (0.3) | Drop-out, absenteeism &academic achievement |
|  | **Cohort B**  (May 2006 to January 2007) |  |  |  |  | (6.5 (0.04) | None |  | 7.9 (0.2) | Drop-out |  | 8.7 (0.2) | Drop-out, absenteeism &academic achievement |
| Mean age for all children | | | | | | (6.5 (0.04) |  |  | 8.4 (0.5) |  |  | 9.3 (0.5) |  |

^†^T0: assessment time-point 0 (2012/2013 academic year), depending upon their birth date child EBD and all other confounders were assessed

^₮^T1: assessment time-point 1 (2013/2014 academic year), all exposures and drop-out (outcome) were assessed simultaneously

^¥^T2: assessment time-point 2 (2014/2015 academic year) outcomes (absenteeism, academic achievement and drop-out) were assessed

**§** standard deviations
