# Supplementary material for: Impact of child emotional and behavioural difficulties on educational outcomes of primary school children in Ethiopia: a population-based cohort study
Source: Child Adolesc Psychiatry Ment Health. 2020 May 16;14:22. doi: 10.1186/s13034-020-00326-6 (PMC7231403; doi:10.1186/s13034-020-00326-6)
Supplement: Supplementary file 2 — Additional file 2. Difference beteen those children who remain and lost to follow-up from T0 to T1 (n = 2090). [file 13034_2020_326_MOESM2_ESM.doc]

# Additional file 2: Difference beteen those children who remain and lost to follow-up from T0 to T1 (n=2090)

| **Characteristics at T0**^†^ | **Had educational information at T1**^₮^ | **Had no educational information at T1** | **χ^2^ P value** |
| --- | --- | --- | --- |
|  | **Number (%)**  **1902 (91.0)** | **Number (%)**  **188 (9.0)** |  |
| Child EBD low SDQ  high SDQ | 1763 (91.2)  131 (87.9) | 170 (8.8)  18 (12.1) | 0.178 |
| Maternal CMD low SRQ  high SRQ | 1813 (91.2)  89 (88.1) | 176 (8.8)  12 (11.9) | 0.299 |
| Maternal literacy literate  Non-literate | 260 (90.6)  1642 (91.1) | 27 (9.4)  161 (8.9) | 0.793 |
| Paternal literacy literate  Non-literate | 1120 (91.7)  706 (91.5) | 102 (8.3)  66 (8.5) | 0.874 |
| Had hunger in last month No  Yes | 1757 (90.8)  145 (93.0) | 177 (9.2)  11 (7.0) | 0.378 |
| Had emergency resource Yes  No | 1135 (92.6)  767 (88.7) | 90 (7.4)  98 (11.3) | 0.002 |
| Roof cover corrugated Iron  Thatched | 653 (91.3)  1249 (90.8) | 62 (8.7)  126 (9.2) | 0.709 |
| Nutritional status non-stunted  Stunted | 1386 (91.4)  507 (89.9) | 131 (8.6)  57 (10.1) | 0.298 |
| Child sex Girl  Boy | 910 (89.6)  996 (91.6) | 106 (10.4)  82 (7.6) | 0.024 |
| Birth order First  Middle or last | 263 (98.5)  1645 (97.9) | 4 (1.5)  35 (2.1) | 0.526 |

^†^T0: assessment time-point 0

^₮^ assessment time-point 1
