# Supplementary material for: Impact of child emotional and behavioural difficulties on educational outcomes of primary school children in Ethiopia: a population-based cohort study
Source: Child Adolesc Psychiatry Ment Health. 2020 May 16;14:22. doi: 10.1186/s13034-020-00326-6 (PMC7231403; doi:10.1186/s13034-020-00326-6)
Supplement: Supplementary file 3 — Additional file 3. Difference beteen those children who do and do not have educational information at T1 (n = 1957). [file 13034_2020_326_MOESM3_ESM.doc]

# Additional file 3: Difference beteen those children who do and do not have educational information at T1 (n=1957)

| **Characteristics at T1**^₮^ | **Had educational information at T2**^¥^ | **Had no educational information at T2** | **χ^2^ P value** |
| --- | --- | --- | --- |
|  | **Number (%)**  **1293 (65.7)** | **Number (%)**  **644 (34.3)** |  |
| Child EBD low SDQ  high SDQ | 1206 (67.5)  69 (61.6) | 584 (32.6)  43 (38.4) | 0.208 |
| Maternal CMD low SRQ  high SRQ | 1148 (65.8)  137 (64.3) | 596 (34.2)  76 (35.7) | 0.662 |
| Maternal literacy literate  Non-literate | 146 (64.0)  1139 (65.9) | 82 (36.0)  590 (34.1) | 0.582 |
| Paternal literacy literate  Non-literate | 759 (67.2)  451 (63.4) | 371 (32.8)  260 (36.6) | 0.100 |
| Had hunger in last month No  Yes | 1222 (65.9)  62 (60.8) | 632 (34.1)  40 (39.2) | 0.288 |
| Had emergency resource Yes  No | 914 (67.8)  371 (60.8) | 433 (32.2)  239 (39.2) | 0.002 |
| Roof cover corrugated Iron  Thatched | 590 (68.9)  695 (63.1) | 266 (31.1)  406 (36.9) | 0.007 |
| Nutritional status non-stunted  Stunted | 985 (72.6)  296 (62.8) | 372 (27.4)  175 (37.2) | <0.001 |
| Child sex Girl  Boy | 611 (65.0)  673 (66.3) | 329 (35.0)  342 (33.7) | 0.544 |
| Middle or last  Birth order First | 1113 (65.9)  172 (64.2) | 576 (34.1)  96 (35.8) | 0.582 |

^₮^ assessment time-point 1

^¥^ assessment time-point 2
