# Supplementary figures and images for: Impact of child emotional and behavioural difficulties on educational outcomes of primary school children in Ethiopia: a population-based cohort study
Source: Child Adolesc Psychiatry Ment Health. 2020 May 16;14:22. doi: 10.1186/s13034-020-00326-6 (PMC7231403; doi:10.1186/s13034-020-00326-6)

# Additional file 4: Graph of SDQ item frequency by sex of the child at T0


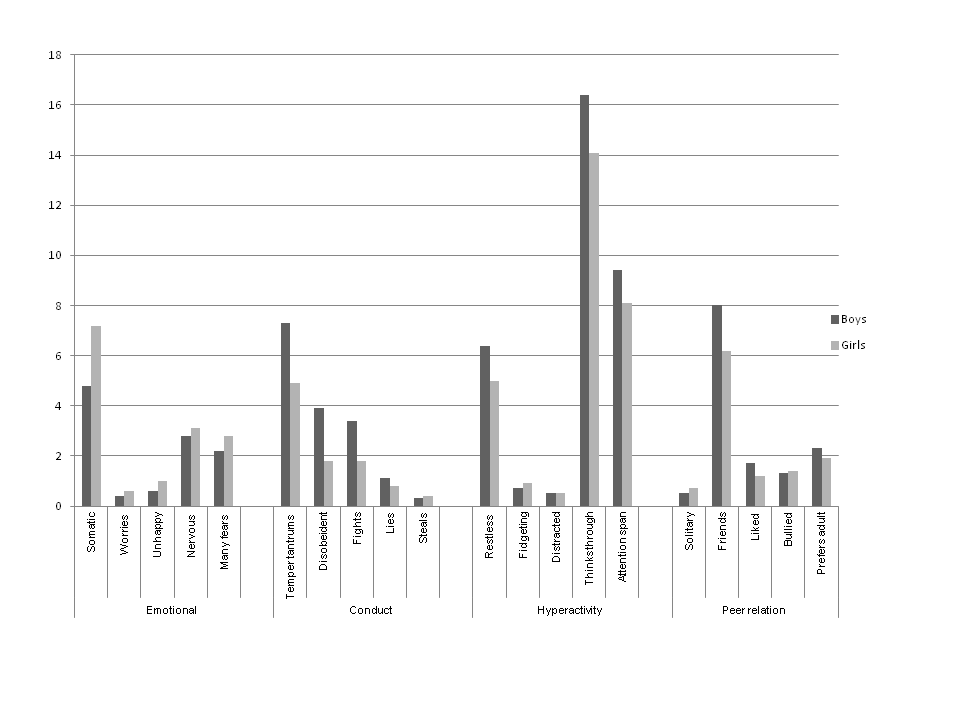

Supplement: Supplementary file 4 — Additional file 4. Graph of SDQ item frequency by sex of the child at T0. [file 13034_2020_326_MOESM4_ESM.doc]
