# Supplementary material for: Impact of child emotional and behavioural difficulties on educational outcomes of primary school children in Ethiopia: a population-based cohort study
Source: Child Adolesc Psychiatry Ment Health. 2020 May 16;14:22. doi: 10.1186/s13034-020-00326-6 (PMC7231403; doi:10.1186/s13034-020-00326-6)
Supplement: Supplementary file 5 — Additional file 5. Maternal report of reasons for school drop-out at T1. [file 13034_2020_326_MOESM5_ESM.doc]

# Additional file 5: Maternal report of reasons for school drop-out at T1

| **Reason for drop-out at T1**^₮^ (n=336) | **Boys (n=201)** | **Girls (n=135)** | **χ^2^ P value** |
| --- | --- | --- | --- |
|  | **Number (%)** | **Number (%)** |  |
| Child refused | 42 (20.8) | 21 (15.7) | 0.150 |
| Child bullied | 52 (25.7) | 32 (23.9) | 0.400 |
| Child misbehaviour /disciplinary action | 10 (4.9) | 1 (0.8) | 0.029^¥^ |
| Child labour | 25 (12.4) | 41 (30.6) | <0.001 |
| Child physical health | 25 (12.4) | 21 (15.7) | 0.285 |
| Economic problems | 13 (6.4) | 3 (2.2) | 0.062^¥^ |
| Distance and unsafe road to school | 12 (5.9) | 6 (4.5) | 0.374^¥^ |
| Parents fear of school environment | 8 (4.0) | 3 (2.2) | 0.295^¥^ |
| Parental report that education not important | 14 (6.9) | 7 (5.2) | 0.348 |

¥Fisher exact

^₮^ assessment time point 1
