# Supplementary material for: Impact of child emotional and behavioural difficulties on educational outcomes of primary school children in Ethiopia: a population-based cohort study
Source: Child Adolesc Psychiatry Ment Health. 2020 May 16;14:22. doi: 10.1186/s13034-020-00326-6 (PMC7231403; doi:10.1186/s13034-020-00326-6)
Supplement: Supplementary file 6 — Additional file 6. Impact of child emotional and behavioural difficulties on absenteeism by cohort. [file 13034_2020_326_MOESM6_ESM.doc]

# Additional file 6: Impact of child emotional and behavioural difficulties on absenteeism by cohort

| Exposures at T1^₮^ | Models | Child school absenteeism at T2^¥^ | | |
| --- | --- | --- | --- | --- |
|  |  | Cohort A (n=293)  Risk Ratio (95% CI) | C-MaMiE cohort (n=464)  Risk Ratio (95% CI) | Cohort B (n=367) Risk Ratio (95% CI) |
| **SDQ sub-scales** | | | | |
| Emotional  Problem (total score) | Crude | 0.99 (0.97, 1.03) | **1.08 (1.05, 1.10)** | **0.97 (0.95, 0.99)** |
|  | Adjusted^†^ | 0.99 (0.96, 1.04) | **1.09 (1.07, 1.12)** | **0.97 (0.94, 0.99)** |
| Conduct Difficulty  (total score) | Crude | 0.99 (0.96, 1.02) | 1.02 (0.99, 1.04) | **0.95 (0.92, 0.97)** |
|  | Adjusted^†^ | 0.98 (0.94, 1.02) | **1.05 (1.02, 1.07)** | **0.97 (0.93, 0.99)** |
| Hyperactivity  Problem (total score) | Crude | 1.01 (0.99, 1.04) | **1.04 (1.02, 1.05)** | 0.98 (0.96, 1.00) |
|  | Adjusted^†^ | 1.00 (0.98, 1.03) | **1.05 (1.03, 1.06)** | 1.01 (0.99, 1.03) |
| Peer relationship  Problem (total score | Crude | 1.03 (0.99, 1.06) | **1.04 (1.01, 1.07**) | **0.93 (0.91, 0.96)** |
|  | Adjusted^†^ | 1.03 (0.99, 1.08) | **1.07 (1.04, 1.11)** | **0.95 (0.91, 0.98)** |
| **SDQ full scale** | | | | |
| Total score | Crude | 1.00 (0.99, 1.01) | **1.02 (1.01, 1.03)** | **0.98 (0.97, 0.99)** |
|  | Adjusted^†^ | 1.00 (0.99, 1.01) | **1.03 (1.02, 1.04)** | **0.99 (0.98, 0.99)** |
| High score (≥14) | Crude | **1.46 (1.23, 1.72)** | **1.36 (1.21, 1.52)** | 0.92 (0.79, 1.08) |
|  | Adjusted^†^ | **1.37 (1.14, 1.65)** | **1.53 (1.34, 1.74)** | 1.12 (0.94, 1.33) |

† maternal age, marital status, maternal and paternal level of literacy, SES, paternal substance use, negative life event, maternal mental health, child sex, birth order and child nutritional status

^₮^ Assessment time-point 1 (2013/2014 academic year),  ^¥^ assessment time-point 2 (2014/2015 academic year)
